# 2,4-Dichloro­benzaldehyde 2,4-dinitro­phenyl­hydrazone

**DOI:** 10.1107/S1600536808020266

**Published:** 2008-07-09

**Authors:** Feng-yu Bao

**Affiliations:** a Department of Applied Chemistry, College of Sciences, Henan Agricultural University, Zhengzhou 450002, People’s Republic of China

## Abstract

The asymmetric unit of the title compound, C_13_H_8_Cl_2_N_4_O_4_, contains two independent but similar and almost planar mol­ecules. An intra­molecular N—H⋯O hydrogen bond is observed in each mol­ecule.

## Related literature

For background, see: Okabe *et al.* (1993 ▶); Ohba (1996 ▶).
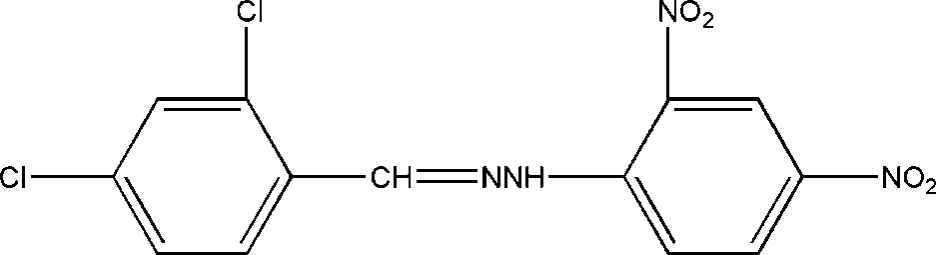


## Experimental

### 

#### Crystal data

C_13_H_8_Cl_2_N_4_O_4_
*M* *_r_* = 355.14
Monoclinic, 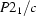

*a* = 13.3814 (7) Å
*b* = 28.9980 (13) Å
*c* = 7.3996 (3) Å
β = 92.422 (4)°
*V* = 2868.7 (2) Å^3^
*Z* = 8
Mo *K*α radiation
μ = 0.48 mm^−1^
*T* = 291 (2) K
0.16 × 0.11 × 0.09 mm

#### Data collection

Bruker SMART APEX CCD diffractometer
Absorption correction: multi-scan (*SADABS*; Bruker, 1998 ▶) *T*_min_ = 0.927, *T*_max_ = 0.958
21827 measured reflections
5060 independent reflections
2794 reflections with *I* > 2σ(*I*)
*R*_int_ = 0.092

#### Refinement

*R*[*F* ^2^ > 2σ(*F* ^2^)] = 0.061
*wR*(*F* ^2^) = 0.187
*S* = 1.02
5060 reflections
415 parameters
H-atom parameters constrained
Δρ_max_ = 0.70 e Å^−3^
Δρ_min_ = −0.36 e Å^−3^

### 

Data collection: *SMART* (Bruker, 1998 ▶); cell refinement: *SAINT* (Bruker, 1998 ▶); data reduction: *SAINT*; program(s) used to solve structure: *SHELXS97* (Sheldrick, 2008 ▶); program(s) used to refine structure: *SHELXL97* (Sheldrick, 2008 ▶); molecular graphics: *SHELXTL* (Sheldrick, 2008 ▶); software used to prepare material for publication: *SHELXTL*.

## Supplementary Material

Crystal structure: contains datablocks global, I. DOI: 10.1107/S1600536808020266/hb2743sup1.cif

Structure factors: contains datablocks I. DOI: 10.1107/S1600536808020266/hb2743Isup2.hkl

Additional supplementary materials:  crystallographic information; 3D view; checkCIF report

## Figures and Tables

**Table 1:** Hydrogen-bond geometry (Å, °)

*D*—H⋯*A*	*D*—H	H⋯*A*	*D*⋯*A*	*D*—H⋯*A*
N6—H6*A*⋯O3	0.86	2.05	2.645 (5)	126
N8—H8*A*⋯O8	0.86	2.03	2.627 (5)	126

**Fig. 1.**
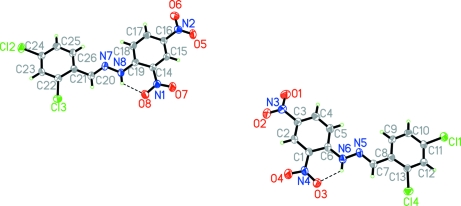
The molecular structure of (I) with displacement ellipsoids drawn at the 30% probability level (arbitrary spheres for the H atoms). The hydrogen bonds are indicated by dashed lines.

## supplementary crystallographic information

### Comment

Several phenylhydrazone derivatives have been shown to be potentially DNA-damaging and are mutagenic agents (Okabe *et al.*,1993). As part of our work in this area, we have synthesized the title compound, (I), and we report its crystal structure here.

The two molecules in the asymmetric unit of (I) are almost planar, the dihedral angles between the the dichlorobenzene ring and the dinitrobenzene ring are 4.4 (2)° and 3.7 (2)°, in the C1 and C14 molecules, respectively. Otherwise, bond lengths and angles agree with those of other dinitrophenylhydrazone derivatives (Ohba, 1996).  Intramolecular N—H···O hydrogen bonds (Fig. 1, Table 1) help to establish these molecular conformations.

### Experimental

2,4-Dinitrophenylhydrazine (1 mmol, 0.198 g) was dissolved in anhydrous methanol, H_2_SO_4_ (98% 0.5 ml) was added to this, the mixture was stirred for several minitutes at 351 K, then 2,4-dichlorobenzyaldehyde (1 mmol, 0.175 g) in methanol (8 ml) was added dropwise and the mixture was stirred at refluxing temperature for 2 h. The product was isolated and recrystallized from DMF, yielding brown blocks of (I) after 6 d.

### Refinement

All H atoms were placed in calculated positions (C—H = 0.93 Å, N—H = 0.86Å) and refined as riding with *U*_iso_(H) = 1.2*U*_eq_(carrier).

### Crystal data

**Table:**

C_13_H_8_Cl_2_N_4_O_4_	*F*_000_ = 1440
*M**_r_* = 355.14	*D*_x_ = 1.645 Mg m^−^^3^
Monoclinic, *P*2_1_/*c*	Mo *K*α radiation λ = 0.71073 Å
Hall symbol: -P 2ybc	Cell parameters from 721 reflections
*a* = 13.3814 (7) Å	θ = 2.5–20.6º
*b* = 28.9980 (13) Å	µ = 0.48 mm^−^^1^
*c* = 7.3996 (3) Å	*T* = 291 (2) K
β = 92.422 (4)º	Block, brown
*V* = 2868.7 (2) Å^3^	0.16 × 0.11 × 0.09 mm
*Z* = 8	

### Data collection

**Table:**

Bruker SMART APEX CCD diffractometer	5060 independent reflections
Radiation source: sealed tube	2794 reflections with *I* > 2σ(*I*)
Monochromator: graphite	*R*_int_ = 0.093
*T* = 291(2) K	θ_max_ = 25.0º
ω scans	θ_min_ = 1.4º
Absorption correction: multi-scan(SADABS; Bruker, 1998)	*h* = −15→15
*T*_min_ = 0.927, *T*_max_ = 0.958	*k* = −34→34
21827 measured reflections	*l* = −8→8

### Refinement

**Table:**

Refinement on *F*^2^	Secondary atom site location: difference Fourier map
Least-squares matrix: full	Hydrogen site location: inferred from neighbouring sites
*R*[*F*^2^ > 2σ(*F*^2^)] = 0.061	H-atom parameters constrained
*wR*(*F*^2^) = 0.187	*w* = 1/[σ^2^(*F*_o_^2^) + (0.0939*P*)^2^] where *P* = (*F*_o_^2^ + 2*F*_c_^2^)/3
*S* = 1.02	(Δ/σ)_max_ = 0.018
5060 reflections	Δρ_max_ = 0.70 e Å^−^^3^
415 parameters	Δρ_min_ = −0.36 e Å^−^^3^
Primary atom site location: structure-invariant direct methods	Extinction correction: none

### Special details

**Table:**

Geometry. All e.s.d.'s (except the e.s.d. in the dihedral angle between two l.s. planes) are estimated using the full covariance matrix. The cell e.s.d.'s are taken into account individually in the estimation of e.s.d.'s in distances, angles and torsion angles; correlations between e.s.d.'s in cell parameters are only used when they are defined by crystal symmetry. An approximate (isotropic) treatment of cell e.s.d.'s is used for estimating e.s.d.'s involving l.s. planes.
Refinement. Refinement of *F*^2^ against ALL reflections. The weighted *R*-factor *wR* and goodness of fit *S* are based on *F*^2^, conventional *R*-factors *R* are based on *F*, with *F* set to zero for negative *F*^2^. The threshold expression of *F*^2^ > σ(*F*^2^) is used only for calculating *R*-factors(gt) *etc*. and is not relevant to the choice of reflections for refinement. *R*-factors based on *F*^2^ are statistically about twice as large as those based on *F*, and *R*- factors based on ALL data will be even larger.

### Fractional atomic coordinates and isotropic or equivalent isotropic displacement parameters (Å^2^)

**Table:**

	*x*	*y*	*z*	*U*_iso_*/*U*_eq_	
C1	0.5169 (3)	0.79565 (15)	0.8938 (6)	0.0525 (11)	
C2	0.4504 (4)	0.76034 (15)	0.8543 (6)	0.0568 (12)	
H2A	0.4681	0.7299	0.8798	0.068*	
C3	0.3594 (4)	0.77030 (16)	0.7782 (6)	0.0567 (12)	
C4	0.3307 (4)	0.81536 (17)	0.7419 (7)	0.0649 (13)	
H4A	0.2670	0.8217	0.6926	0.078*	
C5	0.3956 (4)	0.85013 (17)	0.7786 (6)	0.0630 (13)	
H5A	0.3755	0.8802	0.7532	0.076*	
C6	0.4926 (4)	0.84239 (14)	0.8539 (6)	0.0508 (11)	
C7	0.5828 (4)	0.95479 (14)	0.8532 (6)	0.0522 (11)	
H7A	0.6458	0.9503	0.9085	0.063*	
C8	0.5499 (3)	1.00103 (14)	0.7955 (5)	0.0483 (11)	
C9	0.4563 (3)	1.00735 (15)	0.7104 (6)	0.0526 (11)	
H9A	0.4151	0.9818	0.6913	0.063*	
C10	0.4222 (3)	1.05004 (15)	0.6533 (6)	0.0535 (11)	
H10A	0.3595	1.0534	0.5959	0.064*	
C11	0.4836 (4)	1.08737 (14)	0.6836 (6)	0.0495 (11)	
C12	0.5769 (3)	1.08302 (15)	0.7673 (6)	0.0519 (11)	
H12A	0.6181	1.1086	0.7848	0.062*	
C13	0.6078 (3)	1.03998 (14)	0.8247 (6)	0.0496 (11)	
C14	0.0562 (3)	0.40012 (14)	0.2229 (5)	0.0453 (10)	
C15	−0.0068 (3)	0.43582 (15)	0.1708 (6)	0.0501 (11)	
H15A	0.0142	0.4662	0.1854	0.060*	
C16	−0.1000 (3)	0.42650 (15)	0.0978 (6)	0.0509 (11)	
C17	−0.1325 (4)	0.38143 (16)	0.0780 (6)	0.0572 (12)	
H17A	−0.1972	0.3754	0.0330	0.069*	
C18	−0.0705 (4)	0.34611 (16)	0.1240 (6)	0.0590 (12)	
H18A	−0.0930	0.3160	0.1062	0.071*	
C19	0.0269 (3)	0.35334 (14)	0.1980 (5)	0.0479 (11)	
C20	0.1045 (4)	0.23951 (15)	0.2590 (6)	0.0535 (12)	
H20A	0.1681	0.2440	0.3117	0.064*	
C21	0.0647 (3)	0.19295 (14)	0.2269 (5)	0.0472 (11)	
C22	0.1187 (3)	0.15381 (15)	0.2772 (5)	0.0506 (11)	
C23	0.0818 (4)	0.10936 (14)	0.2470 (6)	0.0517 (11)	
H23A	0.1193	0.0836	0.2818	0.062*	
C24	−0.0113 (4)	0.10474 (15)	0.1644 (6)	0.0513 (11)	
C25	−0.0668 (4)	0.14258 (16)	0.1099 (6)	0.0588 (12)	
H25A	−0.1296	0.1388	0.0528	0.071*	
C26	−0.0283 (4)	0.18613 (15)	0.1409 (6)	0.0552 (12)	
H26A	−0.0657	0.2116	0.1031	0.066*	
N1	0.1514 (3)	0.41294 (14)	0.3070 (5)	0.0577 (10)	
N2	−0.1667 (3)	0.46460 (16)	0.0431 (5)	0.0612 (11)	
N3	0.2910 (3)	0.73221 (16)	0.7304 (6)	0.0708 (12)	
N4	0.6139 (3)	0.78294 (14)	0.9750 (6)	0.0659 (11)	
N5	0.5238 (3)	0.92107 (12)	0.8261 (5)	0.0546 (10)	
N6	0.5553 (3)	0.87810 (12)	0.8820 (5)	0.0541 (10)	
H6A	0.6134	0.8742	0.9336	0.065*	
N7	0.0487 (3)	0.27372 (12)	0.2123 (5)	0.0529 (10)	
N8	0.0870 (3)	0.31688 (11)	0.2431 (5)	0.0532 (9)	
H8A	0.1465	0.3208	0.2890	0.064*	
O1	0.2127 (3)	0.74172 (14)	0.6467 (6)	0.0950 (13)	
O2	0.3142 (3)	0.69353 (13)	0.7743 (6)	0.0985 (13)	
O3	0.6762 (3)	0.81245 (11)	1.0089 (5)	0.0726 (10)	
O4	0.6286 (3)	0.74302 (14)	1.0074 (8)	0.135 (2)	
O5	−0.1387 (3)	0.50380 (12)	0.0700 (5)	0.0796 (11)	
O6	−0.2487 (3)	0.45419 (12)	−0.0264 (5)	0.0833 (11)	
O7	0.1687 (3)	0.45318 (12)	0.3374 (6)	0.0913 (13)	
O8	0.2111 (3)	0.38253 (11)	0.3504 (5)	0.0731 (10)	
Cl1	0.44331 (10)	1.14207 (4)	0.61744 (18)	0.0689 (4)	
Cl2	−0.05792 (11)	0.04957 (4)	0.12777 (17)	0.0700 (4)	
Cl3	0.23487 (9)	0.15728 (4)	0.38772 (18)	0.0705 (4)	
Cl4	0.72292 (10)	1.03677 (4)	0.94132 (19)	0.0724 (4)	

### Atomic displacement parameters (Å^2^)

**Table:**

	*U*^11^	*U*^22^	*U*^33^	*U*^12^	*U*^13^	*U*^23^
C1	0.054 (3)	0.047 (3)	0.057 (3)	0.004 (2)	0.000 (2)	−0.002 (2)
C2	0.061 (3)	0.041 (3)	0.069 (3)	0.001 (2)	0.002 (3)	0.002 (2)
C3	0.055 (3)	0.052 (3)	0.064 (3)	−0.006 (2)	0.004 (2)	0.004 (2)
C4	0.054 (3)	0.060 (3)	0.080 (3)	0.007 (3)	−0.005 (3)	0.012 (3)
C5	0.066 (3)	0.049 (3)	0.073 (3)	0.002 (3)	−0.005 (3)	0.008 (2)
C6	0.061 (3)	0.038 (3)	0.053 (3)	0.000 (2)	0.005 (2)	−0.0013 (19)
C7	0.058 (3)	0.045 (3)	0.053 (3)	0.001 (2)	0.004 (2)	0.002 (2)
C8	0.050 (3)	0.047 (3)	0.048 (2)	0.001 (2)	0.003 (2)	0.0021 (19)
C9	0.055 (3)	0.048 (3)	0.055 (3)	−0.011 (2)	−0.001 (2)	0.000 (2)
C10	0.050 (3)	0.055 (3)	0.055 (3)	0.001 (2)	−0.002 (2)	0.008 (2)
C11	0.060 (3)	0.039 (2)	0.050 (3)	0.004 (2)	0.003 (2)	0.0070 (19)
C12	0.054 (3)	0.045 (3)	0.057 (3)	−0.002 (2)	0.003 (2)	0.006 (2)
C13	0.048 (3)	0.048 (3)	0.052 (3)	−0.002 (2)	−0.005 (2)	0.003 (2)
C14	0.051 (3)	0.044 (2)	0.041 (2)	−0.003 (2)	0.003 (2)	−0.0004 (18)
C15	0.055 (3)	0.044 (3)	0.051 (3)	−0.007 (2)	0.002 (2)	−0.003 (2)
C16	0.052 (3)	0.048 (3)	0.053 (3)	0.000 (2)	0.004 (2)	0.003 (2)
C17	0.051 (3)	0.058 (3)	0.062 (3)	−0.008 (2)	−0.007 (2)	−0.001 (2)
C18	0.063 (3)	0.048 (3)	0.066 (3)	−0.010 (2)	−0.002 (3)	−0.002 (2)
C19	0.053 (3)	0.046 (3)	0.045 (2)	−0.006 (2)	0.003 (2)	0.0022 (19)
C20	0.062 (3)	0.047 (3)	0.052 (3)	−0.004 (2)	0.002 (2)	0.000 (2)
C21	0.056 (3)	0.044 (3)	0.042 (2)	0.004 (2)	0.003 (2)	0.0053 (18)
C22	0.054 (3)	0.055 (3)	0.043 (2)	0.001 (2)	0.002 (2)	−0.0008 (19)
C23	0.059 (3)	0.042 (3)	0.054 (3)	0.006 (2)	−0.002 (2)	0.006 (2)
C24	0.058 (3)	0.051 (3)	0.044 (2)	−0.006 (2)	−0.001 (2)	0.001 (2)
C25	0.058 (3)	0.064 (3)	0.053 (3)	−0.001 (3)	−0.008 (2)	0.003 (2)
C26	0.066 (3)	0.045 (3)	0.054 (3)	−0.004 (2)	−0.003 (2)	0.003 (2)
N1	0.058 (3)	0.049 (3)	0.065 (2)	−0.001 (2)	−0.007 (2)	−0.0022 (19)
N2	0.057 (3)	0.063 (3)	0.062 (2)	0.008 (2)	−0.008 (2)	0.007 (2)
N3	0.059 (3)	0.064 (3)	0.089 (3)	−0.011 (2)	0.000 (3)	0.002 (2)
N4	0.069 (3)	0.039 (2)	0.088 (3)	0.008 (2)	−0.014 (2)	0.006 (2)
N5	0.068 (3)	0.035 (2)	0.060 (2)	0.0043 (19)	0.0047 (19)	0.0060 (17)
N6	0.064 (3)	0.043 (2)	0.056 (2)	0.0031 (19)	0.0001 (19)	0.0027 (17)
N7	0.068 (3)	0.038 (2)	0.053 (2)	−0.0054 (19)	0.0012 (19)	−0.0002 (16)
N8	0.058 (2)	0.039 (2)	0.063 (2)	−0.0008 (18)	−0.0017 (19)	0.0015 (17)
O1	0.072 (3)	0.090 (3)	0.121 (3)	−0.011 (2)	−0.027 (3)	0.002 (2)
O2	0.085 (3)	0.053 (2)	0.157 (4)	−0.008 (2)	−0.013 (3)	0.006 (2)
O3	0.069 (2)	0.052 (2)	0.096 (3)	−0.0040 (18)	−0.019 (2)	−0.0004 (17)
O4	0.094 (3)	0.048 (3)	0.256 (6)	0.001 (2)	−0.075 (4)	0.023 (3)
O5	0.087 (3)	0.046 (2)	0.105 (3)	0.0062 (19)	−0.012 (2)	0.0089 (19)
O6	0.066 (3)	0.084 (3)	0.098 (3)	0.005 (2)	−0.016 (2)	0.007 (2)
O7	0.081 (3)	0.047 (2)	0.142 (4)	−0.0054 (19)	−0.036 (3)	−0.012 (2)
O8	0.070 (2)	0.053 (2)	0.095 (3)	0.0059 (18)	−0.019 (2)	−0.0025 (18)
Cl1	0.0721 (9)	0.0489 (7)	0.0843 (9)	0.0067 (6)	−0.0118 (7)	0.0137 (6)
Cl2	0.0824 (10)	0.0512 (7)	0.0750 (8)	−0.0133 (6)	−0.0150 (7)	−0.0007 (6)
Cl3	0.0564 (8)	0.0704 (9)	0.0833 (9)	−0.0032 (6)	−0.0139 (7)	−0.0004 (6)
Cl4	0.0567 (8)	0.0676 (8)	0.0911 (9)	−0.0022 (6)	−0.0189 (7)	0.0157 (7)

### Geometric parameters (Å, °)

**Table:**

C1—C2	1.379 (6)	C16—N2	1.467 (6)
C1—C6	1.422 (6)	C17—C18	1.352 (6)
C1—N4	1.455 (6)	C17—H17A	0.9300
C2—C3	1.352 (6)	C18—C19	1.408 (6)
C2—H2A	0.9300	C18—H18A	0.9300
C3—C4	1.385 (6)	C19—N8	1.361 (5)
C3—N3	1.468 (6)	C20—N7	1.280 (5)
C4—C5	1.351 (7)	C20—C21	1.467 (6)
C4—H4A	0.9300	C20—H20A	0.9300
C5—C6	1.409 (7)	C21—C26	1.388 (6)
C5—H5A	0.9300	C21—C22	1.388 (6)
C6—N6	1.343 (5)	C22—C23	1.395 (6)
C7—N5	1.267 (5)	C22—Cl3	1.728 (5)
C7—C8	1.469 (6)	C23—C24	1.371 (6)
C7—H7A	0.9300	C23—H23A	0.9300
C8—C13	1.381 (6)	C24—C25	1.376 (6)
C8—C9	1.390 (6)	C24—Cl2	1.734 (4)
C9—C10	1.379 (6)	C25—C26	1.380 (6)
C9—H9A	0.9300	C25—H25A	0.9300
C10—C11	1.372 (6)	C26—H26A	0.9300
C10—H10A	0.9300	N1—O7	1.209 (4)
C11—C12	1.375 (6)	N1—O8	1.224 (5)
C11—Cl1	1.739 (4)	N2—O5	1.211 (5)
C12—C13	1.376 (6)	N2—O6	1.229 (5)
C12—H12A	0.9300	N3—O2	1.204 (5)
C13—Cl4	1.736 (5)	N3—O1	1.226 (5)
C14—C15	1.379 (6)	N4—O4	1.197 (5)
C14—C19	1.422 (6)	N4—O3	1.213 (5)
C14—N1	1.443 (6)	N5—N6	1.373 (5)
C15—C16	1.365 (6)	N6—H6A	0.8600
C15—H15A	0.9300	N7—N8	1.368 (5)
C16—C17	1.384 (6)	N8—H8A	0.8600
			
C2—C1—C6	121.6 (4)	C18—C17—H17A	119.9
C2—C1—N4	117.1 (4)	C16—C17—H17A	119.9
C6—C1—N4	121.3 (4)	C17—C18—C19	122.2 (4)
C3—C2—C1	119.4 (4)	C17—C18—H18A	118.9
C3—C2—H2A	120.3	C19—C18—H18A	118.9
C1—C2—H2A	120.3	N8—C19—C18	120.5 (4)
C2—C3—C4	121.3 (4)	N8—C19—C14	123.5 (4)
C2—C3—N3	118.7 (4)	C18—C19—C14	116.0 (4)
C4—C3—N3	119.9 (5)	N7—C20—C21	117.8 (4)
C5—C4—C3	119.6 (5)	N7—C20—H20A	121.1
C5—C4—H4A	120.2	C21—C20—H20A	121.1
C3—C4—H4A	120.2	C26—C21—C22	116.9 (4)
C4—C5—C6	122.3 (4)	C26—C21—C20	121.2 (4)
C4—C5—H5A	118.8	C22—C21—C20	121.9 (4)
C6—C5—H5A	118.8	C21—C22—C23	122.4 (4)
N6—C6—C5	119.8 (4)	C21—C22—Cl3	121.8 (3)
N6—C6—C1	124.6 (4)	C23—C22—Cl3	115.8 (3)
C5—C6—C1	115.6 (4)	C24—C23—C22	118.1 (4)
N5—C7—C8	118.7 (4)	C24—C23—H23A	120.9
N5—C7—H7A	120.6	C22—C23—H23A	120.9
C8—C7—H7A	120.6	C23—C24—C25	121.5 (4)
C13—C8—C9	116.8 (4)	C23—C24—Cl2	118.3 (3)
C13—C8—C7	122.8 (4)	C25—C24—Cl2	120.2 (4)
C9—C8—C7	120.4 (4)	C24—C25—C26	119.2 (4)
C10—C9—C8	122.5 (4)	C24—C25—H25A	120.4
C10—C9—H9A	118.8	C26—C25—H25A	120.4
C8—C9—H9A	118.8	C25—C26—C21	121.9 (4)
C9—C10—C11	118.0 (4)	C25—C26—H26A	119.0
C9—C10—H10A	121.0	C21—C26—H26A	119.0
C11—C10—H10A	121.0	O7—N1—O8	121.9 (4)
C12—C11—C10	121.8 (4)	O7—N1—C14	119.2 (4)
C12—C11—Cl1	118.4 (3)	O8—N1—C14	118.8 (4)
C10—C11—Cl1	119.8 (4)	O5—N2—O6	124.3 (4)
C11—C12—C13	118.5 (4)	O5—N2—C16	118.7 (4)
C11—C12—H12A	120.8	O6—N2—C16	116.9 (4)
C13—C12—H12A	120.8	O2—N3—O1	123.4 (5)
C12—C13—C8	122.3 (4)	O2—N3—C3	119.0 (5)
C12—C13—Cl4	116.8 (3)	O1—N3—C3	117.6 (4)
C8—C13—Cl4	120.9 (3)	O4—N4—O3	122.3 (4)
C15—C14—C19	121.2 (4)	O4—N4—C1	117.7 (4)
C15—C14—N1	116.4 (4)	O3—N4—C1	120.0 (4)
C19—C14—N1	122.4 (4)	C7—N5—N6	118.1 (4)
C16—C15—C14	119.9 (4)	C6—N6—N5	118.1 (4)
C16—C15—H15A	120.0	C6—N6—H6A	120.9
C14—C15—H15A	120.0	N5—N6—H6A	120.9
C15—C16—C17	120.5 (4)	C20—N7—N8	117.0 (4)
C15—C16—N2	119.7 (4)	C19—N8—N7	117.2 (4)
C17—C16—N2	119.8 (4)	C19—N8—H8A	121.4
C18—C17—C16	120.1 (4)	N7—N8—H8A	121.4

### Hydrogen-bond geometry (Å, °)

**Table:**

*D*—H···*A*	*D*—H	H···*A*	*D*···*A*	*D*—H···*A*
N6—H6A···O3	0.86	2.05	2.645 (5)	126
N8—H8A···O8	0.86	2.03	2.627 (5)	126
